# Supplementary material for: A Nuclear Factor Y-B Transcription Factor, GmNFYB17, Regulates Resistance to Drought Stress in Soybean
Source: Int J Mol Sci. 2022 Jun 29;23(13):7242. doi: 10.3390/ijms23137242 (PMC9266788; doi:10.3390/ijms23137242)
Supplement: Supplementary file 1 [file ijms-23-07242-s001.zip › ijms-1769474-supplementary.pdf]

Table S1. Statistical analysis of four drought tolerance related traits in the association panel and RIL population.

Traits	Population	Mean+SD	Kurtosis	Skewness	CV(%)	Range
GI	Natrual population	0.35±0.18	0.13	0.49	50.57	0.08-1.00
	RIL	0.34±0.24	-0.61	0.27	61.42	0-1.00
MRLI	Natrual population	0.44±0.18	0.29	0.36	41.06	0.11-1.00
	RIL	0.63±0.26	-0.47	-0.65	40.59	0-1.00
BRLI	Natrual population	0.34±0.17	0.31	0.63	50.56	0.06-1.00
	RIL	0.53±0.22	-0.86	-0.18	43.20	0.03-0.97
TRLI	Natrual population	0.51±0.14	1.74	0.6	28	0.25-1.00
	RIL	0.62±0.23	-0.15	-0.74	36.45	0.026-0.98

Table S2 SNP associated with soybean drought tolerance at germination stage identified by GWAS

Trait	Locus name	Chromosome	Alleles	Position	$-Log_{10}(P)$	PVE(%)	Method	Known QTLs or genes
GI	rs42482818	10	G/T	42482818	15.92, 6.61	15.43, 18.96	Blink,MLM	Drought tolerance 6-3(37300089-43004879)
GI	rs23631390	1	G/A	23631390	9.77	10.32	Blink	
GI	rs36378588	2	T/A	36378588	8.26	8.63	Blink	
GI	rs43654888	6	G/T	43654888	7.47	6.56	Blink	
GI	rs27280846	20	G/T	27280846	5.76	5.43	Blink	
BRLI	rs42482818	10	G/T	42482818	23.92, 9.02	16.53, 20.82	Blink,MLM	Drought tolerance 6-3(37300089-43004879)
BRLI	rs50390710	6	C/T	50390710	7.26	6.42	Blink	
BRLI	rs4158130	12	C/A	4158130	6.49	5.31	Blink	
BRLI	rs47334595	10	C/T	47334595	6.21	5.08	Blink	
MRLI	rs42482818	10	G/T	42482818	12.89, 10.51	18.41, 22.62	Blink,MLM	Drought tolerance 6-3(37300089-43004879)
TRLI	rs42482818	10	G/T	42482818	48.05, 17.84	14.19, 21.32	Blink,MLM	Drought tolerance 6-3(37300089-43004879)

Table S3 Genetic linkage map information

Chromosome	Bin marker number	Size(Mb)	Total distance(cM)	Averrage distance(cM)
Chr01	260	55.91	142.31	0.55
Chr02	261	51.65	197.32	0.76
Chr03	291	47.78	149.50	0.51
Chr04	277	49.24	185.19	0.67
Chr05	248	41.93	157.90	0.64
Chr06	281	50.72	371.55	1.32
Chr07	286	44.68	218.53	0.76
Chr08	258	46.99	211.30	0.82
Chr09	281	46.84	130.84	0.47
Chr10	256	50.96	152.31	0.59
Chr11	224	39.17	187.08	0.84
Chr12	278	40.11	148.52	0.53
Chr13	284	44.40	257.03	0.91
Chr14	236	49.71	203.33	0.86
Chr15	244	50.93	140.45	0.58
Chr16	286	37.39	161.18	0.56
Chr17	283	41.90	182.33	0.64
Chr18	252	62.30	200.30	0.79
Chr19	213	50.58	145.65	0.68
Chr20	242	46.77	151.80	0.63

Table S4 QTL mapping underlying soybean drought tolerance at germination stage

QTL	Trait	Chromosome	Position (cM)	Left marker	Right marker	LOD	PVE(%)	Additive effect	Known QTL or genes
qGI1-1	GI	1	120	Chr01_45199493	Chr01_55455938	2.60	9.37	-0.07	Drought index 1-1(45199493-55455938)
qGI10-1	GI	10	99	Chr10_42625169	Chr10_42792796	2.56	9.19	-0.09	Drought tolerance 6-3(42625169-42792796)
qGI14-1	GI	14	74	Chr14_6008788	Chr14_8536163	3.00	13.97	-0.10	
qGI19-1	GI	19	101	Chr19_45082406	Chr19_50007260	3.45	16.82	0.10	
qMRLI1-1	MRLI	1	120	Chr01_45199493	Chr01_55455938	3.24	11.12	-0.10	Drought index 1-1(45199493-55455938)
qMRLI7-1	MRLI	7	161	Chr07_6349115	Chr07_5410219	2.81	9.71	-0.08	
qMRLI7-2	MRLI	7	170	Chr07_5291563	Chr07_3320310	3.10	12.35	-0.10	
qMRLI10-1	MRLI	10	99	Chr10_42625169	Chr10_42792796	4.55	16.47	-0.14	Drought tolerance 6-3(42625169-42792796)
qMRLI16-1	MRLI	16	28	Chr16_33378801	Chr16_33722040	2.6422	10.143	0.0697	
qBRLI7-1	BRLI	7	161	Chr07_6349115	Chr07_5410219	3.1005	15.1643	-0.0848	
qBRLI7-1	BRLI	7	180	Chr07_2699622	Chr07_2535197	3.8167	13.1552	-0.10407	
qBRLI10-1	BRLI	10	103	Chr10_42792796	Chr10_46603218	2.77	10.58	0.07	Drought tolerance 6-3(42625169-42792796)
qBRLI14-1	BRLI	14	74	Chr14_6008788	Chr14_8536163	2.93	14.35	-0.09	
qBRLI15-1	BRLI	15	86	Chr15_15673331	Chr15_17236892	3.21	12.05	0.10	
qBRLI16-1	BRLI	16	28	Chr16_33378801	Chr16_33722040	2.76	10.23	0.06	
qBRLI19-1	BRLI	19	101	Chr19_45082406	Chr19_50007260	2.73	12.48	0.08	ss250269376
qTRLI7-1	TRLI	7	161	Chr07_6349115	Chr07_5410219	2.52	9.09	-0.10	
qTRLI7-2	TRLI	7	180	Chr07_2699622	Chr07_2535197	2.84	7.06	-0.06	
qTRLI10-1	TRLI	10	99	Chr10_42625169	Chr10_42792796	3.64	14.54	-0.11	Drought tolerance 6-3(42625169-42792796)
qTRLI15-1	TRLI	15	86	Chr15_15673331	Chr15_17236892	3.02	10.09	0.08	
qTRLI16-1	TRLI	16	28	Chr16_33378801	Chr16_33722040	3.64	15.56	0.11	
qTRLI19-1	TRLI	19	101	Chr19_45082406	Chr19_50007260	2.92	14.76	0.08	ss250269376

Table S5 The sequence information of *GmNFYB* gene family

Gene	ID	CDS（bp）	Protein（aa）
GmNFYB1	Glyma.02g154000	798	147
GmNFYB2	Glyma.02g300200	738	165
GmNFYB3	Glyma.03g080700	546	174
GmNFYB4	Glyma.03g177700	1079	171
GmNFYB5	Glyma.04G211200	434	221
GmNFYB6	Glyma.05g015100	998	156
GmNFYB7	Glyma.05g193300	624	207
GmNFYB8	Glyma.06G209000	803	156
GmNFYB9	Glyma.07g268100	672	223
GmNFYB10	Glyma.08g001300	666	221
GmNFYB11	Glyma.08g329300	1152	191
GmNFYB12	Glyma.08G141000	1152	191
GmNFYB13	Glyma.09g014100	794	181
GmNFYB14	Glyma.09g046200	483	160
GmNFYB15	Glyma.10g020100	438	145
GmNFYB16	Glyma.10G048900	986	162
GmNFYB17	Glyma.10g192000	1026	174
GmNFYB18	Glyma.10g153500	426	141
GmNFYB19	Glyma.13g003600	986	162
GmNFYB20	Glyma.15g118800	797	171
GmNFYB21	Glyma.15g153900	486	161
GmNFYB22	Glyma.17g005600	681	226
GmNFYB23	Glyma.17g025300	369	122
GmNFYB24	Glyma.17g123600	728	160
GmNFYB25	Glyma.18g077500	826	188
GmNFYB26	Glyma.19g178500	1341	159
GmNFYB27	Glyma.20g000600	507	168
GmNFYB28	Glyma.20g198500	918	173
GmNFYB29	Glyma.20g234900	417	138

Table S6 List of 201 soybean germplasms

Name	Country	Latitude (°N)	Longitude (°W)
HC6	China	22.72	113.33
Hedou	China	22.72	113.33
Mqin2	China	22.72	113.33
Lhang	China	22.72	113.33
Renfenhang	China	22.72	113.33
Pohang	China	22.72	113.33
HC1	China	22.72	113.33
HX1	China	22.72	113.33
HX8	China	22.72	113.33
Liu3	China	23.39	108.17
G0118-1	China	23.39	108.17
Fudou234	China	25.76	117.95
Fall2	China	25.76	117.95
Qan7	China	25.76	117.95
Qan253	China	25.76	117.95
Sgreen	China	25.76	117.95
Gandou4	China	27.17	115.81
G5	China	27.17	115.81
Xinyuqing	China	27.17	115.81

Saugust	China	27.17	115.81
5 months	China	27.17	115.81
Fenchenzao	China	27.17	115.81
Big Zhu	China	27.17	115.81
Rijinqin	China	27.17	115.81
Shaxin	China	27.17	115.81
Short Nidou	China	27.42	111.37
Hangmao	China	27.42	111.37
Changsha2	China	27.42	111.37
8307/8/1	China	30.11	102.76
Gxijangdou	China	30.11	102.76
Chundou8	China	30.11	102.76
84-70	China	31.13	112.14
Honhedou	China	31.13	112.14
Huzimao	China	31.13	112.14
Jinhang35	China	31.13	112.14
Youchun05-4	China	31.13	112.14
Edou7	China	31.13	112.14
Huasedou	China	31.13	112.14
heipidou	China	31.90	116.96
Binhaibaihua	China	32.83	118.88
X12	China	32.83	118.88
Peixianyoudou	China	32.83	118.88
NN30	China	32.83	118.88
Chuxiu	China	32.83	118.88
Sidou2	China	32.83	118.88
Spindinhang	China	32.83	118.88
Tshort	China	32.83	118.88
Theidou	China	32.83	118.88
Xuqandou	China	32.83	118.88
Haidedou	China	32.83	118.88
Pixanpurple	China	32.83	118.88
Shan95	China	33.73	113.30
Hei03687	China	33.73	113.30
YD23	China	33.73	113.30
ZD107	China	33.73	113.30
W8	China	33.73	113.30
Z97196	China	33.73	113.30
ZD17	China	33.73	113.30
ZD11	China	33.73	113.30
Y16	China	33.73	113.30
Y28	China	33.73	113.30
Z92116	China	33.73	113.30
Xudou3	China	33.73	113.30

Huadou20	China	33.73	113.30
Z8516	China	33.73	113.30
Z9525	China	33.73	113.30
Z7016	China	33.73	113.30
Z9805	China	33.73	113.30
Z4066	China	33.73	113.30
Y15	China	33.73	113.30
Y27	China	33.73	113.30
Shichenghongdou	China	33.73	113.30
Keburi	Japan	35.41	139.44
Line1-05-12	Japan	35.41	139.44
Zuoxuan1	China	36.23	118.31
Big black	China	36.23	118.31
Shannin7	China	36.23	118.31
QH28	China	36.23	118.31
QH33	China	36.23	118.31
Hedou12	China	36.23	118.31
Lu4	China	36.23	118.31
QH25	China	36.23	118.31
4seed	China	36.23	118.31
Pindin	China	36.23	118.31
Yhang	China	37.34	84.79
Cateye	China	37.34	100.30
Dahang1	China	37.39	112.24
Jidou9	China	39.19	116.29
JD18	China	39.19	116.29
J17	China	39.19	116.29
Star4	China	39.19	116.29
Datenhei	China	39.19	116.29
Black2	China	39.19	116.29
Nanganqin	China	39.19	116.29
J06B7	China	39.19	116.29
Purple4	China	39.19	116.29
Jin13	China	39.19	116.29
Xamoshidou	China	39.19	116.29
Zhong03-5373	China	40.15	116.28
10C320	China	40.15	116.28
10C373	China	40.15	116.28
10NC831	China	40.15	116.28
Z32	China	40.15	116.28
Z30	China	40.15	116.28
Z03-5355	China	40.15	116.28
Z13	China	40.15	116.28
Early18	China	40.15	116.28

KX3	China	40.15	116.28
Z10	China	40.15	116.28
Z37	China	40.15	116.28
Z8	China	40.15	116.28
Z33	China	40.15	116.28
Z03-5334	China	40.15	116.28
Z03-5363	China	40.15	116.28
Z03-5179	China	40.15	116.28
Z27	China	40.15	116.28
ZJ4032	China	40.15	116.28
Z00-683	China	40.15	116.28
Z05-15	China	40.15	116.28
ZJ4033	China	40.15	116.28
Zhonghuang35	China	40.15	116.28
Zhong95-5388	China	40.15	116.28
Zhong03-5413	China	40.15	116.28
Zhong24	China	40.15	116.28
Hangbaodou	China	40.85	122.00
60hanchang	China	40.85	122.00
Laoxan1	China	40.85	122.00
TF8	China	40.85	122.00
LD23	China	40.85	122.00
LN2	China	40.85	122.00
TD54	China	40.85	122.00
85-140	China	40.85	122.00
Heimodou	China	43.35	126.29
Cmoshi	China	43.35	126.29
JN21	China	43.35	126.29
N989445A	America	43.40	79.25
DN L-13	Canada	43.40	79.25
760	Canada	43.40	79.25
Bayfield	Canada	43.40	79.25
Aika166	Rumania	44.23	26.10
HJ04-528	China	44.49	111.70
HJ423	China	44.49	111.70
Duomala	Yugoslavia	46.30	14.30
Sui20	China	48.29	128.08
HN51	China	48.29	128.08
HF52	China	48.29	128.08
HF47	China	48.29	128.08
HF50	China	48.29	128.08
S04-5804	China	48.29	128.08
Ken15	China	48.29	128.08
Ken18	China	48.29	128.08

Ken14	China	48.29	128.08
Xian2	China	48.29	128.08
HN48	China	48.29	128.08
DN163	China	48.29	128.08
DN49	China	48.29	128.08
HF25	China	48.29	128.08
Heihehangdou	China	48.29	128.08
SN14	China	48.29	128.08
BF9	China	48.29	128.08
FZmoshidou	China	48.29	128.08
Fenshou6	China	48.29	128.08
NF11	China	48.29	128.08
DN47	China	48.29	128.08
HN44	China	48.29	128.08
DN42	China	48.29	128.08
Jishanpu	China	48.29	128.08
DN594	China	48.29	128.08
Dongnong8004	China	48.29	128.08
DN1068	China	48.29	128.08
DN43	China	48.29	128.08
Xian3	China	48.29	128.08
Small Moshi	China	48.29	128.08
Yapiche	China	48.29	128.08
4yellow	China	48.29	128.08
Lonqan	China	48.29	128.08
KB1	China	48.29	128.08
HF35	China	48.29	128.08
NF15	China	48.29	128.08
LP03-11	China	48.29	128.08
LX1	China	48.29	128.08
Heilongjiang41	China	48.29	128.08
HF29	China	48.29	128.08
HF37	China	48.29	128.08
B1361	China	48.29	128.08
B1873	China	48.29	128.08
B4834	China	48.29	128.08
S28	China	48.29	128.08
K04-8579	China	48.29	128.08
S03-3046	China	48.29	128.08
SN25	China	48.29	128.08
S03-3952	China	48.29	128.08
S05-7304	China	48.29	128.08
K30	China	48.29	128.08
K29	China	48.29	128.08

K22	China	48.29	128.08
HN55	China	48.29	128.08
Harvest10	China	48.29	128.08
K4seed	China	48.29	128.08
Heinong33	China	48.29	128.08
Boige du	Germany	52.31	13.20

Table S7 Primers for qRT-PCR analysis

Gene	Primer name	Sequence 5'-3'	Length(bp)	Product Length(bp)	Tm	GC%
Glyma.02g154000	NFYB1-R	ACTTGACCAAAGCTGAGA	18	224	52	44
	NFYB1-F	CGTTAGGAGGAAGGATTT	18		52	44
Glyma.02g300200	NFYB2-R	AGGAATGCGTGTCGGAGTT	19	239	57.6	52.6
	NFYB2-F	ATGATAGGAGGAGGCGGAGG	20		60.5	60
Glyma.03g080700	NFYB3-R	TGTAACATAATGGCGGGAGT	20	290	55.6	45
	NFYB3-F	CGATAGCGGTGAAGGTAAAG	20		55.5	50
Glyma.03g177700	NFYB4-R	AACGAACGGTGGTGATAG	18	241	50.6	50
	NFYB4-F	ATGTTAGCGATAGGGAGGT	19		51.2	47.4
Glyma.04G211200	NFYB5-R	GAGGGTGATACGAAGGGC	18	207	58	61
	NFYB5-F	CATACGTTACAACAAGAGGG	20		53.35	45
Glyma.05g015100	NFYB6-R	GCTAGGATGAACGGTGGA	18	267	57.3	55.6
	NFYB6-F	TAAATGCTAAACGGGAGG	18		52.7	44.4
Glyma.05g193300	NFYB7-R	CGTTTCGGAGTTCATTAG	18	222	52.7	44.4
	NFYB7-F	TGTTGTAGCCTTTGTTCAG	19		53.2	42.1
Glyma.06G209000	NFYB8-R	CTTCCTAAAGCAACAATGAC	20	205	51.3	40
	NFYB8-F	ATCCTAGAACCTGTAGAGCC	20		55.4	50
Glyma.07g268100	NFYB9-R	TGCTTTGGGCAATGAGTA	18	152	52.7	44.4
	NFYB9-F	AGTAGCAAGCGTGGCATA	18		55	50
Glyma.08g001300	NFYB10-R	CTGACAAATGCCAGAGGG	18	198	57.3	55.56
	NFYB10-F	GGTTTTGCTGATGTTGATGTA	21		54.11	38.1
Glyma.08g329300	NFYB11-R	TATTTTCCCTGCACTGACTT	20	257	52.4	40
	NFYB11-F	ACTCTGACACGCACTCCTG	19		53.1	57.9
Glyma.08G141000	NFYB12-R	CGAAGTACACGAAGGAGACG	20	147	55.7	55
	NFYB12-F	CGAAGCCAAGAGTGGTCAT	19		55.5	52.6
Glyma.09g014100	NFYB13-R	TGCGTGTCGGAGTTCATC	18	224	54.8	55.6
	NFYB13-F	CCATAACCCTCATCCCTCT	19		53.5	52.6
Glyma.09g046200	NFYB14-R	TCTTACGAATGCTACCAACA	20	201	51.3	40
	NFYB14-F	AATAGGCTCATTTATCTGGG	20		51.3	40
Glyma.10g020100	NFYB15-R	AATCCTTCCTCCCAACGC	18	139	57.3	55.6
	NFYB15-F	GTCACCATTCACGGTCTT	18		55	50
Glyma.10G048900	NFYB16-R	CAACATCAGCCGCATCAT	18	186	55.1	50
	NFYB16-F	GTGGTCATAGCCCAAAGC	18		53.1	55.6
Glyma.10g192000	NFYB17-R	GCATGGAATGGGTGGAAA	18	147	54	50
	NFYB17-F	TTGAGTTGGTCAGGCAGC	18		56	55
Glyma.10g153500	NFYB18-R	TTGGGCTCTAAGTTCATT	18	175	50.5	38.9
	NFYB18-F	CACGAGTAACCCTAGACAG	19		57.6	52.6
Glyma.13g003600	NFYB19-R	GTCCTCGCTCCTACTTCC	18	211	59.6	61.1
	NFYB19-F	CAATCGTCGTTGATGGTC	18		55	50
Glyma.15g118800	NFYB20-R	TATTTGGTTTGGTGGCATTA	20	171	51.65	35
	NFYB20-F	TATCGGAAGGAACCTGTCT	19		55.41	47.37
Glyma.15g153900	NFYB21-R	CCTCAGAACGCCAAAATC	18	191	53.3	50

	NFYB21-F	CTTCTCATTGGTTCAGCATAGT	22		54.1	40.9
Glyma.17g005600	NFYB22-R	GGAAGACCATAACCGCAGAG	20	231	59.8	55
	NFYB22-F	CAGCACCAAAGTACCCATT	19		55.4	47.4
Glyma.17g025300	NFYB23-R	GGCATCATCAGGGAACAG	18	131	53.3	55.6
	NFYB23-F	AACTCCGACACGCACTCT	18		52.7	55.6
Glyma.17g123600	NFYB24-R	GGAAGAAGCATTAGCAGAGC	20	165	54.6	50
	NFYB24-F	AGACAGGAGGAGTCAGGGTG	20		55.9	60
Glyma.18g077500	NFYB25-R	AGAAGCGCAAGACGATCA	18	212	54.3	50
	NFYB25-F	ACCAGGAGCAGCAGCATA	18		54.2	55.6
Glyma.19g178500	NFYB26-R	TGGTTTATTGGGTAGCAGTC	20	203	52.8	45
	NFYB26-F	TGTGACAAGTTAGTGGGGTG	20		53.5	50
Glyma.20g000600	NFYB27-R	GACAACTACGCTCACCCTC	19	191	52.2	57.9
	NFYB27-F	TCGTCCCTGTAAAACCCT	18		52.3	50
Glyma.20g198500	NFYB28-R	TTTCTCACAAGGTGTTAGTT	20	189	51.65	35
	NFYB28-F	AACCATCTCCTATGTCAGTA	20		53.7	40
Glyma.20g234900	NFYB29-R	TAGAGCGTTGCCGATTGC	18	272	57.3	55.6
	NFYB29-F	CCCTTTCACCTTGCCTGT	18		57.3	55.6
Glyma.12G063400	Actin4-R	GTGTCAGCCATACTGTCCCCATT	23	214	62.2	52.1
	Actin4-F	GTTTCAAGCTCTTGCTCGTAATCA	24		59.8	41.6

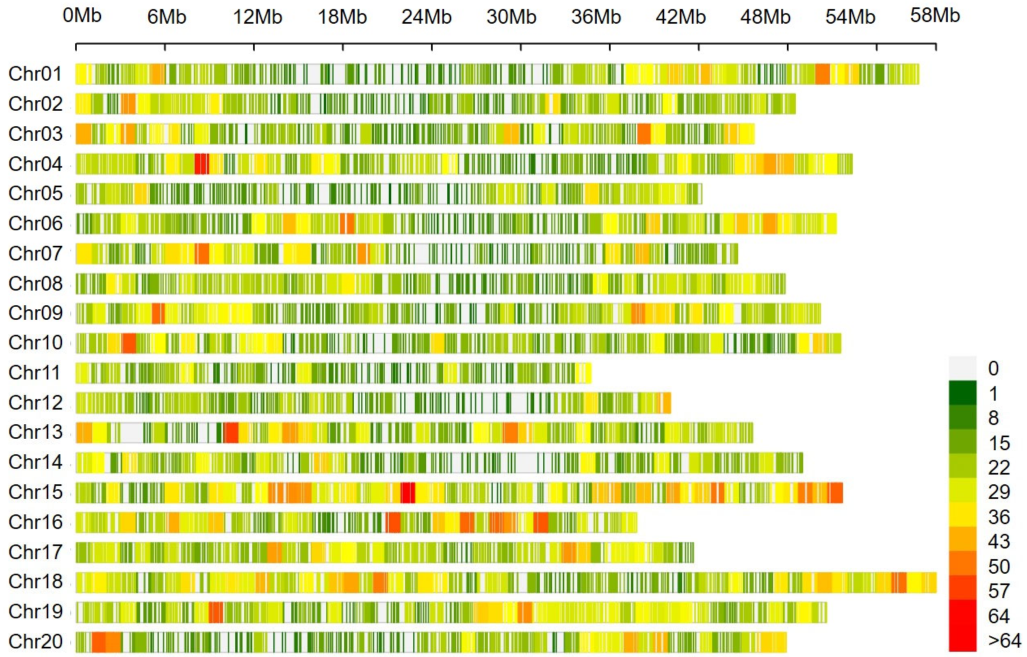

Figure S1. SNP density and distribution for SNP marker across the genome

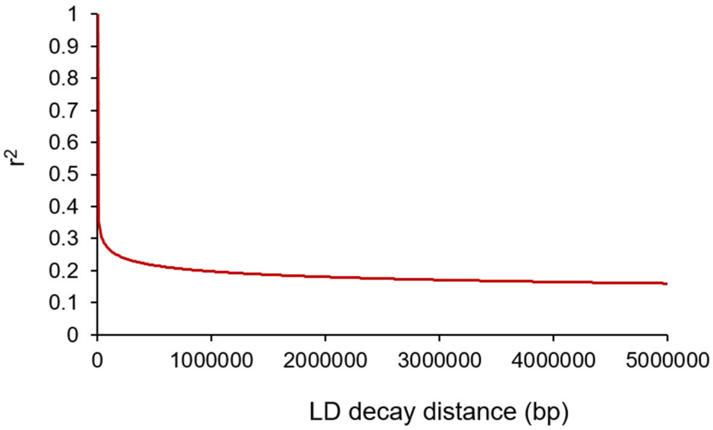

Figure S2. LD Decay pattern of the association panel of 201 soybean accessions

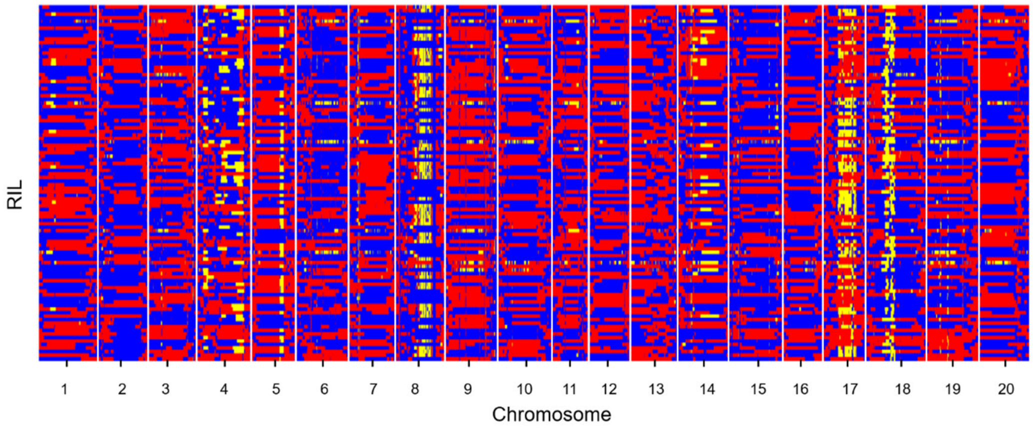

Figure S3. Bin map for 5,241 bin markers across the genome of MH population

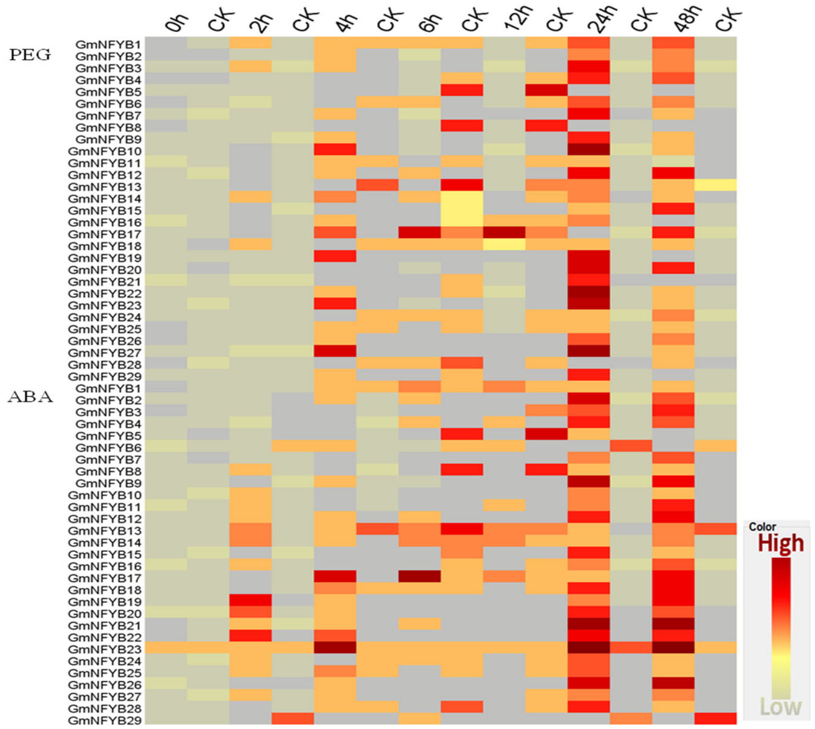

FigureS4. qRT-PCR analysis for differential expression of 29 GmNYFB homologous genes in soybean

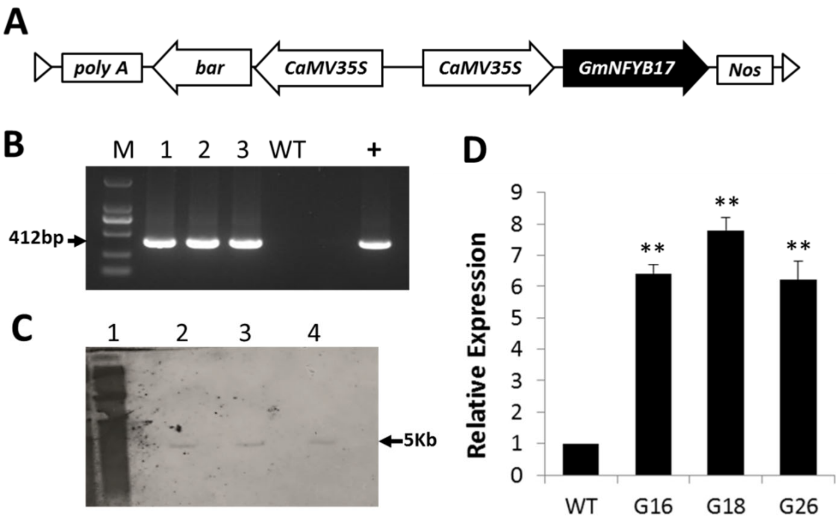

Figure S5. Identification of *GmNFYB17* transgenic soybean lines.
(**A**) T-DNA of overexpression vector pCAMBIA3300-*GmNFYB17*; (**B**) PCR amplification of transgenic soybean lines; M: DL2000, 1-3: lines of G16, G18, G26,

WT: soybean DN50, +:Positive control; (**C**) Southern detection of transgenic plants. 1: Indigested plasmid; 2:G-16 (Hind III enzyme digestion); 3:G-18 (Hind III enzyme digestion); 4:G-26 (Hind III enzyme digestion); (**D**) Detection of gene expression level of GmNFYB17 in transgenic lines by qRT-PCR; Seeds of three transgenic lines (G16, G18 and G26) and non-transgenic control lines (CK) were planted in different plastic bags in greenhouse under normal conditions for 6 weeks. Quantitative real-time PCR was performed to quantify normalized mRNA expression of *GmNFYB17*.
